# Microchannel contacting of crystalline silicon solar cells

**DOI:** 10.1038/s41598-017-08913-y

**Published:** 2017-08-22

**Authors:** James Bullock, Hiroki Ota, Hanchen Wang, Zhaoran Xu, Mark Hettick, Di Yan, Christian Samundsett, Yimao Wan, Stephanie Essig, Monica Morales-Masis, Andrés Cuevas, Ali Javey

**Affiliations:** 1 Department of Electrical Engineering and Computer Sciences, University of California, Berkeley, California, 94720 USA; 2 Materials Sciences Division, Lawrence Berkeley National Laboratory, Berkeley, California, 94720 USA; 3 Research School of Engineering, The Australian National University, Canberra, ACT 0200 Australia; 4 Ecole Polytechnique Federale de Lausanne (EPFL), Institute of Micro Engineering (IMT), Photovoltaics and Thin Film Electronic Laboratory (PVLab), Maladiere 71b, CH-200 Neuchatel, Switzerland

## Abstract

There is tremendous interest in reducing losses caused by the metal contacts in silicon photovoltaics, particularly the optical and resistive losses of the front metal grid. One commonly sought-after goal is the creation of high aspect-ratio metal fingers which provide an optically narrow and low resistance pathway to the external circuit. Currently, the most widely used metal contact deposition techniques are limited to widths and aspect-ratios of ~40 μm and ~0.5, respectively. In this study, we introduce the use of a micropatterned polydimethylsiloxane encapsulation layer to form narrow (~20 μm) microchannels, with aspect-ratios up to 8, on the surface of solar cells. We demonstrate that low temperature metal pastes, electroless plating and atomic layer deposition can all be used within the microchannels. Further, we fabricate proof-of-concept structures including simple planar silicon heterojunction and homojunction solar cells. While preliminary in both design and efficiency, these results demonstrate the potential of this approach and its compatibility with current solar cell architectures.

## Introduction

All photovoltaic cells require a set of outer metal electrodes to convey the current and voltage developed within the cell to the external circuit. Such contacts must balance a number of competing issues, including contact shading, line resistance, contact resistance, technological limitations and cost (particularly for Ag contacts). For more than 30 years, the metal contacts of silicon solar cells have been made by the simple process of printing metal pastes through patterned screens or stencils. Although there has been significant advancement in this technology over this period^1^, today’s minimum finger width and maximum aspect-ratios (height/width) are still limited to around 40 μm and 0.5, respectively^2, 3^. In addition, the use of such pastes commonly requires high temperatures and can only make low resistance contact to certain surfaces and doping concentrations. Due to these limitations, significant research has been devoted towards the development of alternative contacting schemes which target narrow fingers, high aspect-ratios and low resistance interfaces with different c-Si surfaces.

Among the most notable alternatives to the conventional technology are electrochemical plating^4–6^, print-on-print^3^, aerosol fine line printing^7, 8^, and metal - wrap - through contact approaches^9^, some of which have already been transferred or trialed in cell production lines. However, these approaches still suffer from relatively modest aspect-ratios and are only effective on certain surfaces. One interesting approach to contact formation, with limited development within the field of c-Si PV^10–13^, is the use of microchannels as a template to define the contacts. Within the broader scientific community microchannels have been widely utilized, most prominently in the fabrication of biomedical and lab-on-a-chip type microfluidic devices^14–17^. Generally speaking, this involves the formation of micron-scale channels for passing minute quantities of fluids or gasses and can now be fabricated with a range of materials including silicon, glass, polymers and metals. Here we introduce a versatile approach based on polydimethylsiloxane (PDMS) microchannels which can form high aspect-ratio contacts for solar cells with low optical width and resistance. Compared to previous microchannel contacting studies, we favor a simple approach, focusing on the use of 10–40 μm wide microchannels, which can be introduced to a number of existing solar cell technologies. We highlight the versatility of this technology by demonstrating contact deposition within the microchannel using a range of simple approaches.

## Results and Discussion

### Microchannel contacted solar cell concept

**Figure 1:**
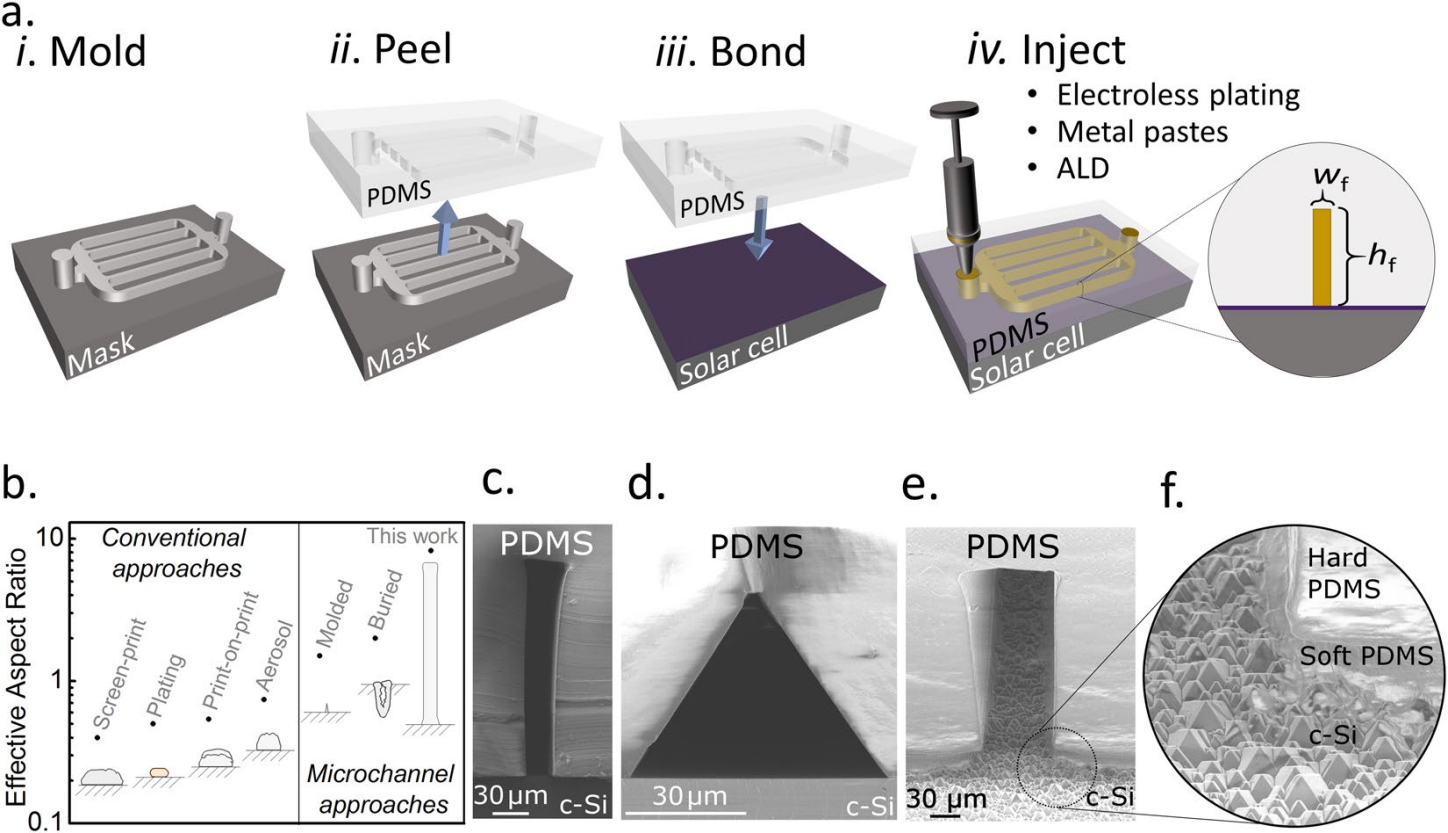
Microchannel contacted solar cell concept. (**a**) Basic fabrication procedure for microchannel contacted solar cells, (**b**) Comparison of aspect-ratio for conventional and microchannel based solar cell contacts, the individual figures approximately reflect their size relative to each other. Cross sectional SEM images of (**c**) high aspect-ratio microchannel (90° cross section); (**d**) triangular microchannel (90° cross section); (**e**) textured surface bonding (45° cross section); and (**f**) magnified bonding to textured surface (45° cross section).

**Figure 2:**
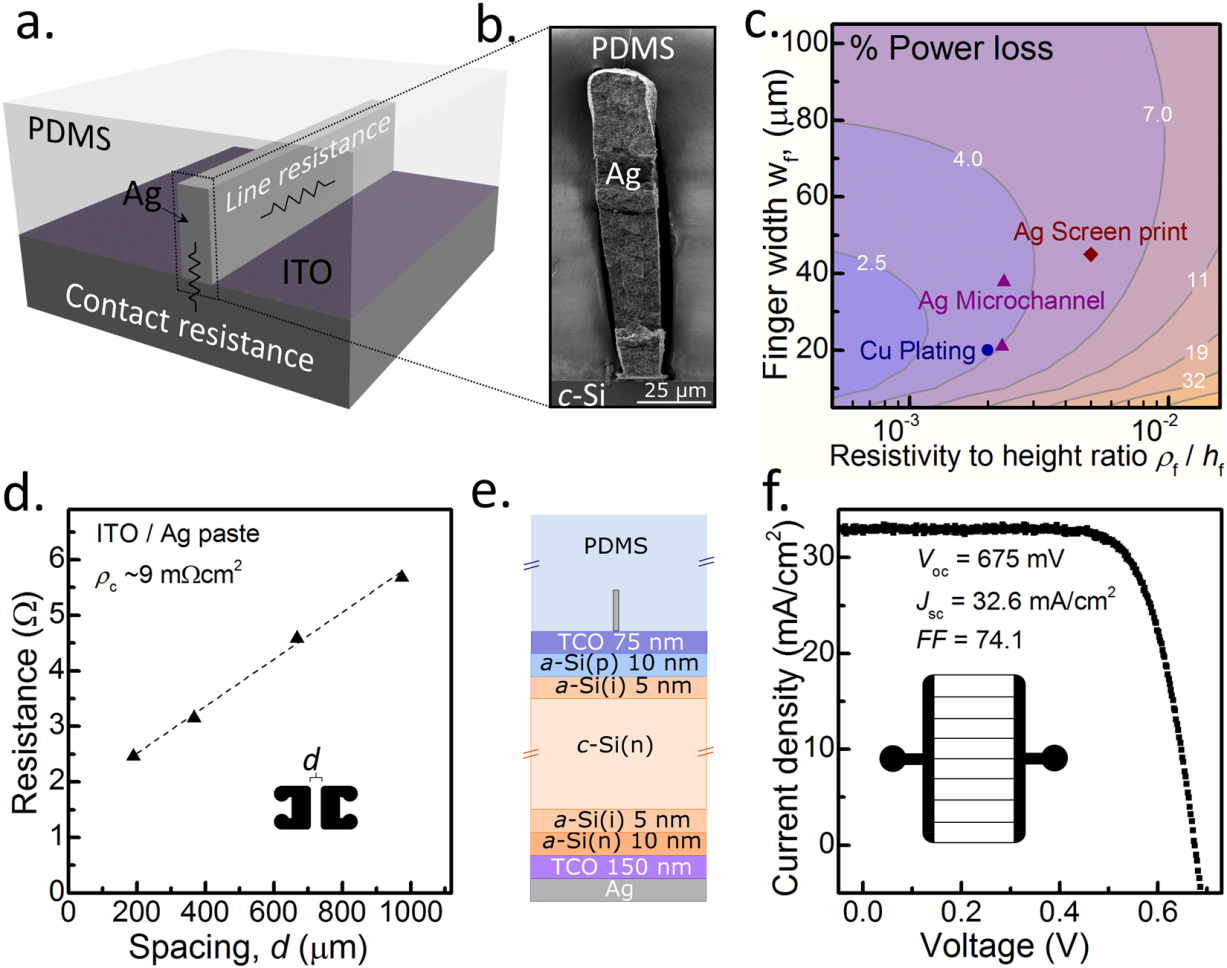
Ag paste injection within the microchannel. (**a**) Schematic of microchannel contacted ITO layer showing the contributions of line and contact resistance, (**b**) cross section of Ag paste filled microchannel layer showing complete filling of the microchannel, (**c**) Simulations of the power loss percentage of an otherwise ideal solar cell as a function of the front contact finger width and the resistivity to height ratio, (**d**) contact resistivity extraction of ITO/Ag paste interface (Inset shows TLM pad design). Planar SHJ solar cell (**e**) layer design; and (**f**) 1 sun *JV* results (the inset of (f) shows the solar cell front grid design).

**Figure 3:**
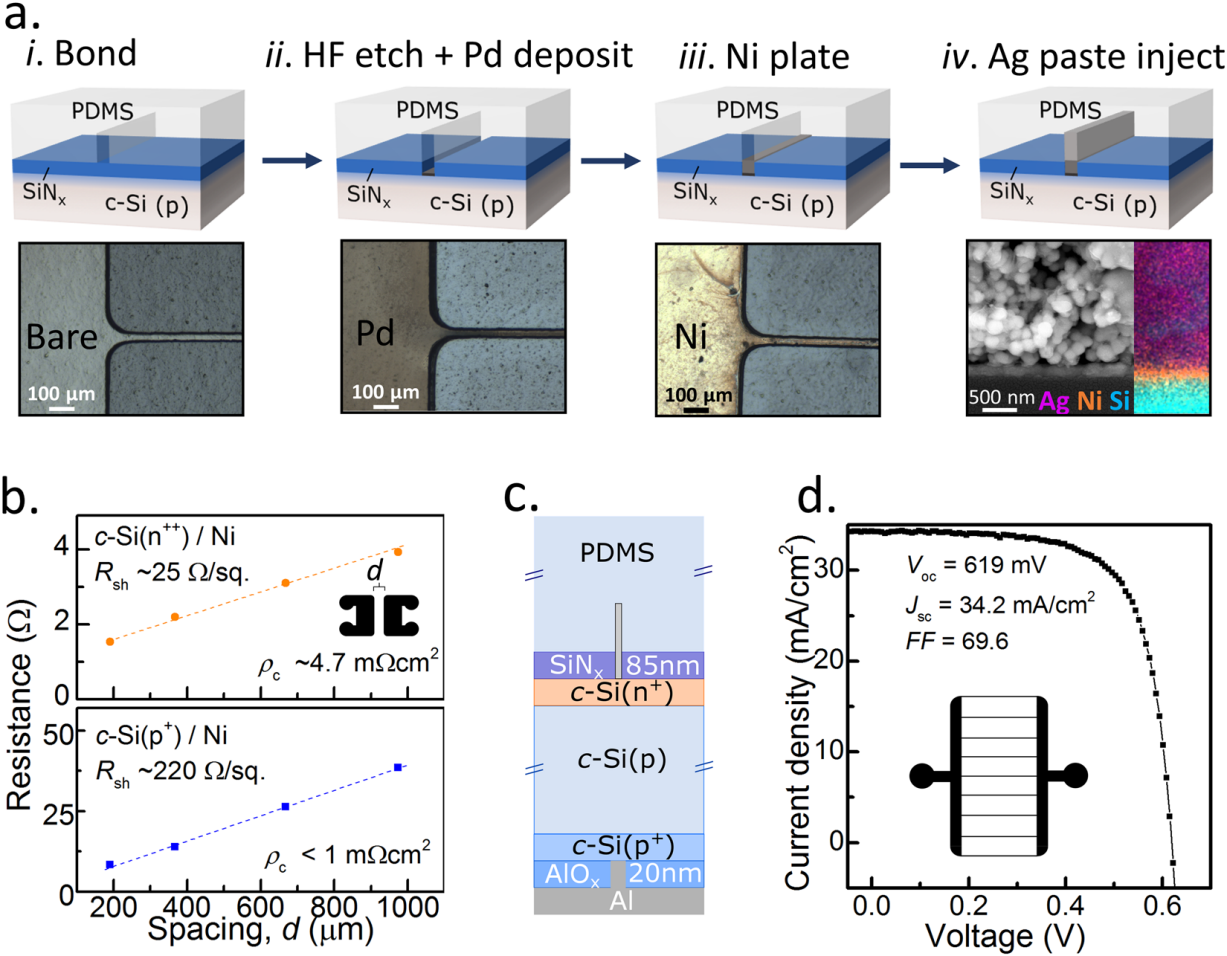
Electroless plating within the microchannel. (**a**) Nickel electroless plating procedure used in this study, for steps *i*-*iii* a top-view optical microscope image is provided, and for step *iv* a cross sectional SEM image with accompanying EDX mapping of local Ag, Ni and Si concentration is included. (**b**) Contact resistivity extraction of Ni plated layer on n^++^ (top) and p^+^(bottom) surfaces. Planar heterojunction solar cell (**c**) layer design; and (**d**) 1 sun *JV* results.

**Figure 4:**
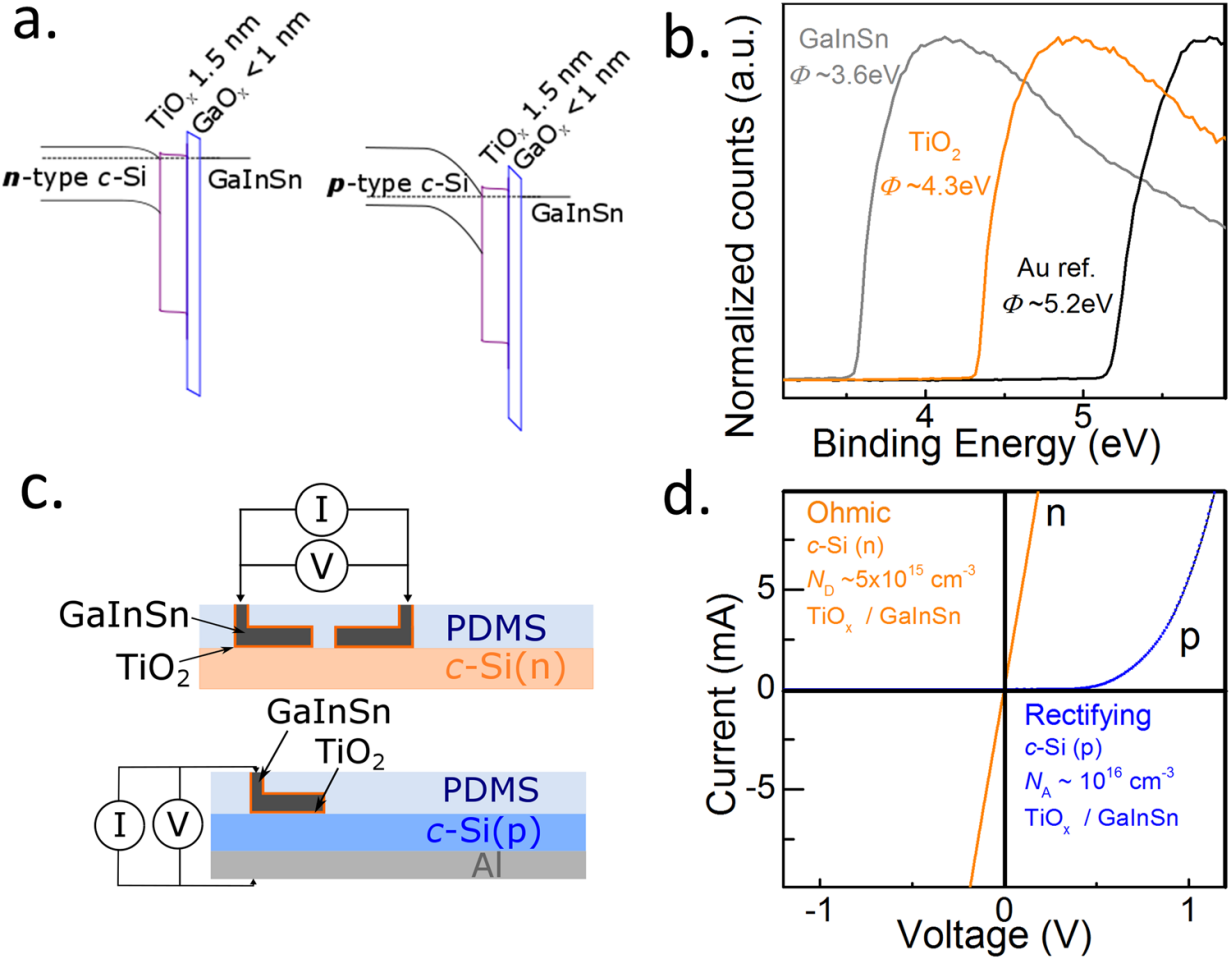
Carrier-selective contacts within the microchannel. (**a**) Idealized band structure of TiO_x_/GaO_x_/GaInSn electron contacts, (**b**) XPS secondary electron cutoff energy showing work function of GaInSn and TiO_x_ layers alongside Au reference. (**c**) Test structure diagrams of microchannel electron selective contacts made to n-type (top) and p-type (bottom) silicon wafers. (**d**) *IV* behavior of selective contact structures shown in (**c**).

Figure 1 shows the basic concept of the microchannel contacted solar cell developed in this work. As depicted in panel a), the microchannels are formed by first curing PDMS over a micropatterned mold. Following this the PDMS is peeled from the mold and bonded permanently to the front surface of a solar cell, leaving microchannel voids on the cell’s surface. This procedure allows the PDMS to also function as a cell encapsulant, with a number of potential benefits over conventional EVA based encapsulants, including greater transparency^18, 19^, an advantage already acknowledged in projections of the International Technology Roadmap for Photovoltaics (ITRPV)^2^. The PDMS microchannel voids on the cell surface are then used as a template with which to define the solar cell contacts via three different techniques. These three techniques are highlighted in later figures of this paper: Ag paste injection for narrow, low resistivity fingers (see Fig. 2); Ni electroless plating for low resistivity Ohmic contacts with heavily doped c-Si (see Fig. 3); and atomic layer deposition for selective-contact formation (see Fig. 4).

One of the main advantages of this microchannel approach lies in the ability to easily form narrow, high aspect-ratio channels. Figure 1b shows current best-in-class metal contact finger widths and aspect-ratios, for a number of existing commercially relevant technologies^3–5, 7, 20, 21^, plotted beside three microchannel based approaches^11, 22^, the last of which is the novel approach developed in this study. It can be seen that, assuming the metal contact fills most of the channel void, this approach offers the potential for very high aspect - ratios (up to 8) and smaller contact widths (~20 μm) than all the conventional approaches. While simplicity has been favored in this work, with a more specialized procedure these two characteristics can be improved further, to aspect - ratios of ~20 or sub-micron widths if desired^23, 24^. It is also possible to control the shape of the microchannel, as shown in the scanning electron microscope (SEM) images in Figures 1c and d, offering benefits of reduced finger shading compared to conventional contacts, a factor already highlighted in previous studies^10, 22^. While planar surfaces are primarily used in this proof-of-concept study, by incorporating an additional partially cured PDMS layer, bonding can also be achieved on textured surfaces, as shown in the SEM images of Figures 1e and f. However, further work will be required to transfer the contacting practices described below to textured surfaces.

### Metal paste injection into the microchannel

To realize the full advantages of the microchannel approach it is necessary to be able to fill most of the channel cross section with a high conductivity material which is also able to form a low resistivity contact to the underlying solar cell. As depicted in Figure 2a, the first approach explored in this study was to inject and then cure a commercially available low temperature Ag paste (manufactured by Heraeus), with a slight dilution to decrease its pre-curing viscosity. Unlike conventional Ag paste, which requires a high temperature spike anneal (~800 °C), this paste can be used with a curing temperature of ~200 °C. Figure 2b shows a cross sectional SEM image of a channel after injection and curing, confirming that the channel can be filled using this approach. An upper-limit estimate of the cured paste resistivity *ρ*_f_ is made by injecting into a single microchannel of known length, height and width and measuring the total resistance between the inlet and outlet vias after curing. Using this approach a value of *ρ*_f_ ~2.2 × 10^−5^ Ωcm is obtained, similar to values measured using standard low temperature screen-printing^6^. It is worth noting that the length and width of this microchannel was ~2.2 cm and ~20 μm indicating that Ag paste injection can be made through long distances of narrow microchannel. It is also possible that this process could be enhanced by chemically pretreating the microchannel walls to increase capillary action or by further optimizing the paste constitution.

To understand the potential advantage of this approach, Figure 2c presents simulations of power loss percentage of an ideal silicon solar cell as a function of the front contact finger characteristics. These simulations account for both finger shading and resistive losses (for more details on the simulation see the experimental section)^8^. The simulations show that the power loss can be reduced by minimizing finger width at the same time as decreasing the finger bulk resistivity to height ratio (*ρ*_f_/*h*_f_). Superimposed on this plot are values of best-in-class Cu plated and low temperature Ag screen printed contacts^6^, as well as two values for Ag microchannel fingers (of different width) fabricated in this study. The comparison demonstrates that the microchannel approach already provides a promising path to lower metal grid power losses for solar cells. Further still, existing microchannel demonstrations within the literature suggest that with further development, contacts with characteristics at the optimum point in Figure 2c could be fabricated^24^.

As mentioned above, a second important characteristic of the electrode is to ensure that it has a low resistivity interface with the underlying solar cell. In this instance, the contact resistivity *ρ*_c_ between the injected Ag paste and a sputtered indium tin oxide (ITO) thin film was measured by the transfer length method (TLM). ITO is a common transparent conductive oxide (TCO), utilized for the sunward electrode in many different absorber type solar cells, and is chosen here as a demonstration of the general applicability of the microchannel approach.

The TLM test structure was fabricated by injecting the Ag paste into a series of channels forming pairs of pads separated by different distances; an example of a single pad set is shown in the inset of Figure 2d. A upper limit *ρ*_c_ of ~9 mΩcm^2^ is obtained for the Ag/ITO interface, which is slightly higher than other values reported in the literature^6^, though not prohibitive to its usage. It should be mentioned that due to the fabrication restrictions of these microchannel TLM structures (with rounded edges and without the possibility of edge isolation), they do not fully satisfy the requirements of accurate TLM measurements. As such, the obtained *ρ*_c_ should be taken as an approximate value only.

To demonstrate the proof-of-concept for the Ag paste filled microchannel, planar silicon heterojunction (SHJ) solar cells are fabricated using microchannel contacts on the front-side. SHJ cells are generally characterized by the positive attributes of low processing temperatures and high open circuit voltages *V*_oc_. However, they suffer from parasitic absorption in the front amorphous silicon and transparent conductive oxide (TCO) layers. The use of microchannel templated contacts offers several additional benefits with the SHJ architecture, including processing temperature compatibility and the ability to use more transparent (less conductive) TCOs with narrower, more regularly spaced metal fingers.

The layer structure of the planar SHJ cell utilized in this study is sketched in Figure 2e and the front microchannel grid design is shown in the inset of Figure 2f. The front grid consists of an inlet and outlet microchannel bus-bar connected by 9 thin microchannel fingers of ~4 mm in length separated by a pitch of ~1.5 mm. A representative 1 sun, current density - voltage (*JV*) curve for this cell is also provided in Figure 2e, showing an efficiency above 16%–a promising result given the limitations this of basic cell configuration. More specifically, the low current density is expected from the planar surface of the silicon wafer and the open circuit voltage *V*_oc_ is also limited to some degree by the small cell area (0.7 cm^2^). Regardless of this, the results demonstrate the compatibility of the microchannel approach with the SHJ fabrication procedure.

### Electroless plating within the microchannel

Extending the above approach to contact crystalline silicon directly is not possible due to a prohibitively high *ρ*_c_ between the low temperature Ag paste and c-Si. To address this issue, an electroless Ni plating process was developed (shown in Fig. 3a), to be used as an interlayer to facilitate contact between the c-Si and the Ag finger. After permanently bonding the PDMS layer to the wafer surface, a combined channel etch and Pd particle deposition (used as a catalyst for the Ni plating) is performed by flowing an aqueous solution of PdCl_2_ 1 g/L, HCl (37% stock concentration) 1 ml/L, HF (48% stock concentration) 100 ml/L through the microchannel. In the case where the wafer surface is covered by a thin insulating layer, such as silicon nitride (SiN_x_), this approach can be used to selectively etch the insulator away within the microchannel only. After a 10-minute forming gas anneal at 250 °C, an electroless Ni plating solution is passed through the channel at 80–100 °C to deposit an ~150 nm layer. This solution was prepared by adjusting an aqueous solution of NiCl_2_ 30 g/L, NaPO_2_H_2_ 20 g/L, Na_3_C_6_H_5_O_7_ 12.5 g/L, CH_3_COONa 5 g/L to a pH of ~9.5 using NH_4_OH. A final 5 minute, 300 °C anneal in forming gas is used to achieve a low resistivity interface before injecting the Ag paste to fill up the rest of the microchannel. In this instance, Ag paste was utilized purely to reduce the finger line resistivity but it could easily be substituted with a less expensive, low temperature Cu paste^25^. Cu diffusion into the silicon substrate is unlikely to be an issue due to the Ni interlayer, which is known to be an excellent Cu diffusion barrier. It should be noted that the parasitic absorption from the PDMS layer is expected to increase after annealing at temperatures above ~250 °C^26, 27^. However, due to the short anneal times used in this study, this increase in absorption was minimized to an upper-limit loss of ~0.4 mA/cm^2^ as calculated from transmission measurements of the PDMS layer before and after the anneal steps (not shown).

Figure 3a *iv* shows a SEM image and an adjacent energy-dispersive X-ray  (EDX) mapping of the resultant interface, indicating the formation of a uniform layer of Ni in-between the c-Si and Ag layers. To investigate the *ρ*_c_ of the c-Si/Ni interface for heavily boron and phosphorus doped silicon surfaces, a set of TLM microchannels (similar to those discussed above) were prepared on n^++^ (25 Ω/sq) and p^+^ (220 Ω/sq) dopant diffused surface regions. As can be seen in Figure 3b *ρ*_c_ values of ~4.7 mΩcm^2^ and <1 mΩcm^2^ are measured on n^++^ and p^+^ surfaces respectively. It is significant that a *ρ*_c_ on the mΩcm^2^ scale can be obtained on the 220 Ω/sq p^+^ surface given the current trend of lightening surface doping diffusions to improve the recombination current prefactor *J*_0_ in the non-contacted, surface passivated regions. Again, given the fabrication limitations of these TLM structures the extracted *ρ*_c_ values should be used as a guide only.

To demonstrate the proof - of - concept of this Ni plating approach further, conventional planar n^++^/p/p^++^ homojunction cells (the structure of which is shown in Fig. 3c), are fabricated with microchannel contacts on the front surface, using the approach depicted in Figure 3a. A front microchannel grid, identical to that used for the SHJ cells in Figure 2 above, is used. The 1 sun *JV* cell results provided in Figure 3d show a conversion efficiency of close to 15% for a ~0.7 cm^2^ area cell, again mainly limited by the basic cell design. In particular, the cell efficiency is limited by the planar front surface and Auger recombination in the heavily doped surface region. The low measured fill factor of ~70% is most likely due to poor adhesion quality between the Ag paste and the Ni plated layer. Nonetheless, these results demonstrate the potential of Ni plating within the microchannel as a means to contact dopant diffused homojunction solar cells.

### Carrier selective contacts within the microchannel via ALD

As a final extension to this microchannel contacting technology, the use of carrier-selective contacts on lightly doped crystalline silicon surfaces is explored. This topic has received considerable attention within the PV community in recent years, owing to its simplicity and high efficiency potential^28–31^. Carrier selectivity is typically achieved by introducing an asymmetry in conductivity for the two carriers at the contacted surface. In this study, we combine the use of both extreme work function values and uneven band offsets to achieve this asymmetry. To do so, we implement a TiO_x_/liquid GaInSn metal electron selective contact—a potential band structure of which is shown in Figure 4a. The large TiO_x_ /c-Si valence band offset promotes the blocking of holes and the low work function of the eutectic metal alloy GaInSn encourages downward band bending, increasing the electron concentration at the c-Si surface. The low work function of the GaInSn, and to a lesser degree the TiO_x_, is confirmed using secondary electron cutoff (SEC) X-ray photoelectron spectroscopy (XPS) as shown in Figure 4b. Core level XPS analysis (not shown) also confirmed the presence of an unavoidable GaO_x_ layer (<1 nm) covering the surface of the GaInSn^32^. Therefore, the abovementioned GaInSn SEC measurement incorporates the contribution of the thin GaO_x_ surface layer. The electronic behavior of this heterocontact is measured using simple two pad structures as shown in Figure 4c. The TiO_x_ layer of ~1.5 nm is deposited in the microchannel via atomic-layer-deposition at 120 °C. By using an appropriately designed deposition recipe this technique was confirmed to deposit uniformly throughout the channel. Following this, GaInSn was injected to fill the remaining channel volume. It is worth mentioning that due to the liquid form of the GaInSn alloy, this approach may have additional benefits if applied to flexible solar cells. The dark current-voltage *IV* behavior of this contact stack on n and p-type c-Si is shown in Figure 4d. As expected, Ohmic behavior is observed between two TiO_x_/GaInSn contacts on n-type c-Si. Conversely, rectifying behavior is found on p-type c-Si (the Al layer used as the rear contact is known to form Ohmic contact on p-type c-Si). These results confirm the potential of this contact strategy as an electron selective-contact. This demonstration also suggests that two opposite selective-contacts could be placed in adjacent, closely spaced microchannels, allowing facile fabrication of interdigitated-back-contact (IBC) solar cell structures.

## Conclusions

In this study, we have introduced PDMS microchannels as a versatile means of templating narrow contacts with aspect - ratios up to 8. We have highlighted three different, low temperature deposition techniques as a general tool-box for contacting within the microchannels, based around Ag paste injection (for low resistivity, high aspect-ratio fingers), Ni electroless plating (for low resistivity contact to highly doped c-Si) and the deposition of carrier-selective contacts by ALD. These techniques are demonstrated in simple proof-of-concept contact and solar cell test structures, with promising properties. Finally, we emphasize that the low temperature microchannel concept presented here could be easily extended to other solar cell architectures and absorber types with further optimization, using a range of microchannel formation materials (including EVA)^33, 34^, and other contact deposition techniques^35^.

## Methods

### PDMS microchannel fabrication and bonding

Molds in this study were fabricated using a standard SU8 photolithography procedure^36^. These are made hydrophobic by vapor treatment for >1 hour in Trichloro(1H,1H,2H,2H-perfluorooctyl) silane vapor. The PDMS is formed from a commercially available two-part mixture (Sylgard 184), poured over the SU8 mold and cured at 90 °C for 20 minutes on the hotplate. The PDMS is peeled from the mold and inlet/outlet vias are made through the entire PDMS layer using a biopsy punch. After an isopropanol rinse and short O_2_ plasma treatment, the PDMS layer is placed on the desired surface and bonded at 90 °C for 15 minutes. For some surfaces an adhesion promotor, (3-Aminopropyl)triethoxysilane (APTES) in methanol (0.05 ml/ml), was applied to the non-PDMS layer prior to O_2_ plasma treatment. Bonding to the textured surface (shown in Fig. 1e and f) was facilitated through the use of a thin partially cured layer of PDMS on the bottom of a micropatterned PDMS slab, which was applied to the textured surface before fully curing (a dilute HF etch was used to remove isolated spots of PDMS residue within the microchannel). The mold for the triangular channel presented in Figure 1c is achieved by forming a SiN_x_ hardmask on a Si wafer and subjecting it to an anisotropic 50% KOH etch.

### Contact test structures

ITO/Ag paste contact test structures were fabricated by bonding a series of microchannel pad pairs separated by 4 different spacings on ITO (~80 nm 100 Ω/sq, RF sputtering, AJA international) coated glass. A diluted commercially available low temperature Ag paste was injected into the microchannels using a syringe. Following injection, these were cured at 200 °C for 30 minutes. For smaller channels a more dilute paste was favored allowing a second injection and curing process forming well filled channels. Line resistivity samples were fabricated in a similar manner using single 2.2 cm long microchannels of different widths bonded to a high resistivity Si wafers.

For the Ni electroless plating contact resistivity samples, microchannel pad sets were bonded to silicon wafers with n^++^ (25 Ω/ sq) or p^+^ (220 Ω/sq) surface diffusions, achieved using a dedicated clean quartz furnace and POCl_3_ and BBr_3_ sources. To deposit the Ni layer, initially the channel was etched and Pd particles were deposited on the silicon surface by injecting a solution of PdCl_2_ 1 g/L, HCl (37% stock) 1 ml/L, HF (48% stock) 100 ml/L in the channel, and allowing it to sit for ~30 sec. The Pd particles help to catalyze the Ni electroless deposition process. After rinsing, the sample is annealed at 250 °C for 10 minutes in forming gas (5% H_2_, 95% N_2_), following which it is retreated with the HF-PdCl_2_-HCl solution. After rinsing again, electroless Ni plating solution was injected through the channel at 80–100 °C using a syringe pump to deposit a ~150 nm layer. The aqueous plating solution of NiCl_2_ 30 g/L, NaPO_2_H_2_ 20 g/L, Na_3_C_6_H_5_O_7_ 12.5 g/L, CH_3_COONa 5 g/L was adjusted to a PH of ~9.5 using NH_4_OH. The sample is then rinsed with DI water and annealed at 300 °C for 5 minutes in forming gas to improve the contact. The change in transparency of the PDMS layer after the forming gas anneal steps was assessed by comparing the transmission of PDMS sheets after curing with that of a sheet subjected to both the 250 °C and 300 °C anneal step (320–1100 nm, PerkinElmer Lambda 950). The maximum *J*_sc_ loss is calculated using the AM1.5G spectrum.

The carrier selectivity test structures were fabricated on 1–2 Ωcm n and p-type samples. Following bonding of pad sets the TiO_x_ layer is deposited via thermal atomic-layer-deposition using alternating pulses of titanium tetraisopropoxide (TTIP) and water at 120 °C. Following this the eutectic metal GaInSn is injected into the microchannel using a syringe. The Ohmic contact to the p-type sample is achieved by thermal evaporation of Al on a freshly HF etched surface.

### Solar cell proof-of-concept structures

The SHJ cells were fabricated on double side polished ~3 Ωcm n-type Si wafers. After HF treatment, intrinsic (~5 nm) and then doped (~12 nm) amorphous silicon layers were deposited via plasma-enhanced-chemical-vapor-deposition (PECVD) at 200 °C (KAI-M). Following this, a ~75 nm front ITO layer (~55 Ω/sq) and an ITO/Ag (150 nm /150 nm) rear contact stack were deposited by sputtering. A PDMS layer with a microchannel front grid design (0.7 cm^2^) is bonded to the surface and the Ag paste is injected as described in the main text.

The homojunction cells were fabricated on 1 Ωcm p-type wafers, with n^++^ (42 Ω /sq) and p^++^ (50 Ω/sq) surface diffusions on the front and rear-side, respectively. These surface diffusions were achieved in dedicated clean quartz furnaces using POCl_3_ and BBr_3_ sources respectively. A low pressure chemical vapor deposition (LPCVD) SiN_x_ hardmask was used to protect the front surface during the BBR_3_ diffusion, before being removed prior to the POCl_3_ diffusion. The front-side is passivated using 85 nm of SiN_x_ deposited using PECVD (Roth and Rau AK 400), and the rear is deposited with ~20 nm of AlO_x_ deposited via ALD (Beneq TFS 200, Trimethylaluminium and water at 200 °C). Rear dots covering approximately 1% of the surface area are opened using photolithography and ~200 nm of Al is deposited as the rear contact via thermal evaporation. The PDMS front grid microchannel is bonded and Ni is deposited selectively in the channel using the procedure described in the main text above. Following this, Ag paste is injected into the channel and cured to reduce the grid resistance.

Light *JV* measurements are taken at 1 sun (100 mW/cm^2^, 25 °C, AM1.5 G spectrum) using a SolarLight solar simulator calibrated with a Thorlabs silicon photodiode (NIST calibrated). To define the cell area from a larger front ITO pad, an aperture mask with an opening of ~0.7 cm^2^ was used. In some instances, the PDMS had to be partially cut away to allow better contact to the metal layers.

### Other characterization

Cross sectional scanning electron micrographs and accompanying energy-dispersive X-ray mappings were taken using a FEI Quanta FEG 250 SEM with a Bruker QUANTAX EDS spectrometer. X-ray Photoelectron spectroscopic analysis was performed on thin layers of TiO_x_ and GaInSn deposited on n-type silicon wafers using a Kratos Axis Ultra DLD spectrometer, with a monochromatic Al Kα source (excitation energy of 1486.7 eV). A bias of 9 V was applied to assist in extraction of secondary electrons at the cutoff edge, which was subtracted in the final data shown. Instrument work function calibration was performed against a gold reference argon sputtered *in situ*, which was also used as the work function reference displayed in Figure 4b.

### Simulations

The simulations were performed using the simple analytical finger power loss analysis described in ref. 8 assuming current and voltage values of an ideal silicon solar cell. The finger spacing and length was set at 2.08 mm and 2.6 cm based off a 3 bus bar front grid design.

The datasets generated during and/or analysed during the current study are available from the corresponding author on reasonable request
